# HEARING LOSS, HEARING AIDS, AND CHANGE IN FUNCTIONAL STATUS: THE ATHEROSCLEROSIS RISK IN COMMUNITIES (ARIC) STUDY

**DOI:** 10.1093/geroni/igae098.1075

**Published:** 2024-12-31

**Authors:** Pablo Martinez Amezcua, Sahar Assi, Erica Twardzik, Nicholas Reed, Laura Scow, Anna Kucharska-Newton, B Gwen Windham, Priya Palta

**Affiliations:** Johns Hopkins University, Baltimore, Maryland, United States; Johns Hopkins University, Baltimore, Maryland, United States; University of Michigan, Ann Arbor, Maryland, United States; Johns Hopkins University, Baltimore, Maryland, United States; Johns Hopkins University, Baltimore, Maryland, United States; University of North Carolina at Chapel Hill, Chapel Hill, North Carolina, United States; University of Mississippi Medical Center - The MIND Center, Jackson, Mississippi, United States; University of North Carolina, Chapel Hill, North Carolina, United States

## Abstract

Hearing loss is common among older adults and is linked to poor physical function, but whether hearing aid use may slow physical function declines remains unknown. We investigated the association between hearing loss and functional status in older adults and modifying effects of hearing aid use. We used data from the Atherosclerosis Risk in Communities (ARIC) cohort study to examine cross-sectional (2016-2017) and longitudinal relationships (2016-2022) after adjustment for sociodemographic and health-related characteristics. Hearing loss (none, mild, moderate/severe) was based on the better-hearing ear’s pure tone average at speech frequencies. Hearing aid use was self-reported. Participants self-reported (any difficulty, regardless of level, vs. none) difficulties with activities of daily living (ADLs), instrumental activities of daily living (IADLs), and heavier tasks (lifting heavy objects, kneeling, going up stairs without stopping). Cross-sectionally, among 3,142 community-dwelling older adults (mean age=79, 58% female, 21% Black), moderate or greater hearing loss was associated with increased difficulties in ADLs (odds ratio [OR]=1.27, 95% CI 1.02, 1.58), IADLs (OR=1.34, 95% CI 1.05, 1.71), and heavier tasks (OR=1.29, 95% CI 1.04, 1.62) relative to no hearing loss. Longitudinally, over an average follow-up of 1.9 years, moderate or greater hearing loss was associated with higher odds of reporting any functional difficulty (OR=1.14, 95% CI 1.05, 1.24). Hearing aid use did not modify the associations. These findings emphasize the importance of hearing loss, its adverse effects on physical function in aging populations, and the need for comprehensive interventions beyond hearing aids for adults with hearing loss.

